# Physical therapy treatments for low back pain in children and adolescents: a meta-analysis

**DOI:** 10.1186/1471-2474-14-55

**Published:** 2013-02-02

**Authors:** Inmaculada Calvo-Muñoz, Antonia Gómez-Conesa, Julio Sánchez-Meca

**Affiliations:** 1 Department of Physiotherapy, Faculty of Medicine, Espinardo Campus, University of Murcia, Murcia 30100, Spain; 2 Department of Basic Psychology and Methodology, University of Murcia, Murcia, Spain

**Keywords:** Physical therapy, Effectiveness, Low back pain, Children, Adolescents, Meta-analysis

## Abstract

**Background:**

Low back pain (LBP) in adolescents is associated with LBP in later years. In recent years treatments have been administered to adolescents for LBP, but it is not known which physical therapy treatment is the most efficacious. By means of a meta-analysis, the current study investigated the effectiveness of the physical therapy treatments for LBP in children and adolescents.

**Methods:**

Studies in English, Spanish, French, Italian and Portuguese, and carried out by March 2011, were selected by electronic and manual search. Two independent researchers coded the moderator variables of the studies, and performed the effect size calculations. The mean effect size index used was the standardized mean change between the pretest and posttest, and it was applied separately for each combination of outcome measures, (pain, disability, flexibility, endurance and mental health) and measurement type (self-reports, and clinician assessments).

**Results:**

Eight articles that met the selection criteria enabled us to define 11 treatment groups and 5 control groups using the group as the unit of analysis. The 16 groups involved a total sample of 334 subjects at the posttest (221 in the treatment groups and 113 in the control groups). For all outcome measures, the average effect size of the treatment groups was statistically and clinically significant, whereas the control groups had negative average effect sizes that were not statistically significant.

**Conclusions:**

Of all the physical therapy treatments for LBP in children and adolescents, the combination of therapeutic physical conditioning and manual therapy is the most effective. The low number of studies and control groups, and the methodological limitations in this meta-analysis prevent us from drawing definitive conclusions in relation to the efficacy of physical therapy treatments in LBP.

## Background

The high prevalence of low back pain (LBP) in children and adolescents has been demonstrated in various epidemiological studies [1-4]. According to the literature, the lifetime prevalence of LBP in children and adolescents varies from 9% [5] to 69% [6]. The prevalence of LPB increases considerably between the ages of 12 and 18 [7-10].

The factors which are known to be significantly associated with LBP in childhood and adolescence are: lifestyle factors [6,11,12], physical factors [13,14], school-related factors [15,16] and psychosocial factors [17]. Furthermore, various authors have stated that LBP in adolescence is associated with LBP in the future [13,18,19]. Children and adolescents with LBP often have a disability [20-23]. Watson et al. [21] found that 94% of children with LBP had some kind of disability, the most common one being difficulty in carrying their school bags. Trevelyan and Legg [23] found that 13.9% of 245 children and adolescents had LBP and 98% of these claimed to have some limitation in activities of daily life. Although about 33.6%-56% of adolescents with LBP have limitations for some activities, Pellise et al. [1] found that 9 out of every 10 adolescents reporting LBP can be considered healthy, while in a 10% of them LBP can be considered as a symptom of a multidimensional health problem.

The high prevalence of LBP in children and adolescents and the predictive value that LBP in adolescence has on suffering LBP as an adult, have led to preventive and therapeutic physical therapy treatments [24-28] for LBP being carried out in the child and adolescent stage [29-31].

Preventive treatments have been carried out over the last three decades. These treatments cover postural hygiene content [24,25], the practice of physical therapy exercises [26,27] and the promotion of physical activity [28].

In adults, the treatment of LBP has been investigated extensively [32-35], and there is evidence that physical therapy treatment with exercise, back school and manual therapy are effective methods for reducing pain and functional limitations in adults [36-38]. However, therapeutic treatments for LBP in children and adolescents are more recent, and the treatments applied consist of back education [30,31], exercise [29,31], manual therapy [30] and therapeutic physical conditioning [29,30]. These interventions are primarily aimed at reducing the prevalence and intensity of LBP and disability, although it is unknown which treatment is most effective in this population.

To date, there has been no meta-analysis on the effectiveness of physical therapy treatments for LBP in children and adolescents. The purpose of our research is to review the empirical evidence, by applying a meta-analysis on the differential effectiveness of physical therapy treatment for LBP in children and adolescents to determine whether the treatment is beneficial for pain, disability and other outcome variables. We are also interested in studying the influence that the treatment, the participants, the context, and the methodological and extrinsic variables have on effect sizes.

Based on the literature on the subject, we hypothesize that the type of treatment used will be an influential variable on the effect size magnitude.

## Methods

### Selection of the studies

In order to be included in our meta-analysis, studies had to meet the following criteria: (a) Physical therapy methodologies of treatment for LBP; (b) The study could include one or more different treatment groups, with or without a control group, but all had to have pretest and posttest measures; (c) Studies could be published or unpublished; (d) Studies had to have a group design; single-case designs were excluded; (e) Studies had to have the statistical data necessary for calculating the effect sizes; (f) Years considered: no restrictions regarding the beginning date, but the study had to be published or carried out by March 2011; (g) Languages: English, Spanish, French, Italian and Portuguese; (h) Age: 6 to 18 years; (i) LBP in the whole sample or part of it; (j) Studies in which subjects in the sample presented LBP that was secondary to the following features were excluded: serious spinal pathologies or deformities, neurological conditions which alter motor tone.

### Search procedure

In order to select the studies that met the selection criteria the following databases were consulted: Cochrane Library, ISI Web of Knowledge, Medline, PEDro y LILACS. The search period went up to March 2011. The key words were combined as follows: [adolescent* or child* or youn* or school*] and [back pain or low back pain or back complaint* or back care] and [treatment or intervention or education or postural hygiene or posture education or back function or physiotherapy or ergonomics or physical therapy or exercise or exercise therapy or management or chiropractic or physical fitness or movement techniques or acupuncture or tens or massage or spinal manipulation or rehabilitation or back school or conservative or manual therapy or recuperation]. In the Medline search, this combination of key words was applied with the following additional characteristics: all years, all languages, all publication types, all citation subsets, all child (0–18 years), species (humans), all genders, all databanks, all statues, and with the field tags: topic. An example of the full electronic search strategy for Medline is provided in Additional file 1.

The electronic search identified 1,337 references which were reviewed to determine whether they met the selection criteria. The main reasons for deleting these studies were because the participants in the samples were adults (about 40%), applying pharmacological treatments for LBP (about 35%), or by other reasons (about 25%). Specialist electronic journals, conference papers and doctoral theses were also consulted. Finally, the references of the studies we identified were reviewed and contact was made with research experts in the field.

The result of the search process allowed us to select 8 articles that met the selection criteria, which meant a total of 16 groups, of which 11 were treatment groups and 5 were control groups. The Additional file 2 describes the process of selection and exclusion of studies. The 16 groups formed a total sample of 334 subjects at the posttest (221 participants in the treatment groups and 113 in the control groups).

### Coding of the studies

In order to analyze the heterogeneity of the study results, the characteristics that could be related to the effect sizes were coded. The moderator variables were grouped into three categories according to the recommendations of Lipsey [39]: substantive variables (of treatment, context and subject), methodological variables and extrinsic variables.

The following treatment characteristics were coded: (a) Type of treatment (back education, exercise, manual therapy and therapeutic physical conditioning); (b) Type of back education, (acquisition of knowledge, posture training habits, body awareness training); (c) The teaching mode of back education (theoretical, practical); (d) The type of exercise (stretching, strengthening, breathing, posture correction, balance exercises, functional exercises, warm-up, relaxation, coordination, stabilization); (e) The type of manual therapy (mobilization, manipulation, massage); (f) The type of therapeutic physical conditioning (walking, running, cycling, swimming); (g) The duration of the treatment (in weeks); (h) The intensity of treatment (number of hours per week of treatment per subject); (i) The treatment magnitude (total number of hours of treatment per subject); (j) The number of sessions established; (k) The homogeneity of the treatment; (l) The inclusion of home exercise program; (m) The inclusion of a follow-up program; (n) The use of external agents (subjects that are not part of the therapeutic group, who are not professionals but who have an influence and can support the subjects in achieving their therapy goals); (o) The presence of relatives or sports coaches acting as co-therapists (who continue or carry out the treatment in other areas); (p) The mode of treatment (therapist, previously trained co-therapist, subject with therapist, unsupervised subject); (q) The type of training (group, individual or mixed); (r) The use of informed consent. With regards to the characteristics of the therapists the following variables were coded; (s) The number of therapists; (t) Whether or not the study’s authors agree with the therapists; (u) The therapist’s training (physical therapist, or other); (v) The therapist’s experience (high, medium, low or mixed).

The following subject characteristics were coded: (a) The average age of the sample (in years); (b) The gender of the sample (% of males); (c) The level of physical activity of the subjects during the study (low, moderate, regular); (d) The average duration of pain (in months); (e) Whether they have received previous treatment or not; (f) The presence or absence of other disorders. The following contextual characteristics were coded: (a) The country and (b) The place where the treatment took place (university, clinic, health centre / day care centre, hospital, school, sports centre, mixed).

Regarding the methodological characteristics, the following were included: (a) The assignment of subjects to treatment groups (random versus non random); (b) The type of control group (active versus inactive), when there was one; (c) The longest follow-up (in months); (d) The sample size; (e) The attrition at the post test; (f) The attrition at the follow-up; (g) The methodological quality of the study on a scale of 0 to 8 points, which is the sum of the scores of eight quality items (random assignment, type of control group, sample size, attrition, intent-to-treat analysis, evaluator blinding, homogeneous assessment, and inter-rater reliability) was analyzed according to the criteria of van Tulder [40] adapting some items to our work. Finally, the following extrinsic characteristics were coded: (a) The year of publication of the study; (b) The training of the first author (physical therapist or other) and (c) The publication source (published versus unpublished). To ensure the maximum possible objectivity, a coding manual was created that specified the rules followed in encoding each of the characteristics of the studies. The coding of certain characteristics required complex decisions to be made. In order to test the appropriateness of these decisions, we conducted a reliability study of the coding process and two researchers independently coded all of the studies. For the quantitative moderator variables the coding reliability was calculated using the intra-class correlation coefficient (ICC), while for the qualitative moderator variables Cohen’s kappa coefficient was applied. The ICC was 0.988 (range: 0.886 to 1) and the kappa coefficient was 0.977 (range 0.792 to 1), which is highly satisfactory, as proposed by Orwin [41]. The inconsistencies between the coders were resolved by consensus and the coding manual was corrected when the cause of these inconsistencies was due to an error in it. The coding manual can be obtained from the corresponding author.

### The effect size

Given the lack of control groups in this area of research, we chose to use the group as the unit of analysis instead of the comparison between a treatment group and a control group. The standardized mean change index, *d*, was used as the effect size index. *d* is defined as the difference between the means of the pretest and the posttest, divided by the standard deviation of the pretest [42]. Positive *d* indexes indicated an improvement from pretest to posttest. The within-study sampling variance of the *d* index was calculated following Morris (2000). To calculate this sampling variance, the pretest-posttest scores correlation is needed. As the studies did not report it, then a common value of 0.5 was assumed for all of them. In order to check whether the value of the correlation coefficient can affect the meta-analytic results, a sensitivity analysis was carried out consisting into calculate the sampling variances of the effect sizes by assuming *r* values of 0.2 and 0.8.

The *d* index is methodologically weaker than comparing a treatment group with a control group, since it is more prone to bias due to factors such as the mere passing of time, the effects of history or spontaneous remission. However, it is the only viable alternative if not all studies include a control group. Nevertheless, since we obtained *d* indices for the treatment and control groups, the difference between the two enabled us to estimate the net effects of treatment.

With the purpose of checking whether the standardized pretest-posttest difference might be offering a biased estimate of the treatment effects, we also calculated the between-groups standardized mean difference with the five studies that included a control group. In this case, the effect size in each study was calculated as the difference between the standardized mean change of the treatment and the control groups. The sampling variance of this new effect size index was the sum of the sampling variances of the treatment and control within-study effect sizes. A comparison between the mean within-group effect size and the mean between-groups effect size enabled us to examine the potential existence of an overestimation of the treatment effects with the within-group effect sizes.

The results of each study were classified according to outcome measure: (a) pain, (b) disability, (c) flexibility, (d) endurance and (d) mental health. The different results were also classified according to the measurement type: self-reports and clinician assessments. In addition, an overall effect size was calculated in each single group by averaging the effect sizes for the different outcome measures reported in the study. For each outcome measure and measurement type both a within- and a between-groups *d* index was calculated.

In order to check the reliability of the effect size calculations, two independent researchers carried out the calculations for all of the studies, reaching an intra-class correlation coefficient of 0.987 (range: 0.882-1), which is also highly satisfactory.

### Statistical analysis

Separate meta-analyses were carried out for each combination of outcome measures and measurement types. In order to give more weight to the effect sizes with larger sample sizes, each effect size was weighted by the inverse variance.

In each meta-analysis and assuming a random-effects model, we calculated a weighted mean effect size together with its confidence interval for the treatment groups and control groups separately. The same calculations were done with the between-groups effect sizes. Following Cohen [43], we interpreted the effect sizes of 0.20, 0.50, and 0.80 as representing low, medium and high effect magnitudes, respectively. The comparison between the treated and control groups was carried out by weighted ANOVA so that the *Q*_b_ test enabled us to check if there were significant differences between the average effects of the treated and control groups. To test the influence of other moderator variables, weighted ANOVAs and simple meta-regressions were used for the qualitative and continuous variables, respectively. The residual heterogeneity variance was estimated by the method of moments proposed by DerSimonian and Laird. There are other heterogeneity variance estimators proposed in the literature. In order to check whether the selection of the variance estimator can affect the meta-analytic results, the analyses were repeated by using a variance estimator based on the restricted maximum likelihood (REML) method.

To test the differential effectiveness of the different types of treatment we applied a mixed-effects multiple meta-regression model.

Finally, we checked whether publication bias could be a bias source in the effect size estimates in our meta-analysis [44].

All statistical analyses were performed using SPSS macros created by David B. Wilson [45] and the programs REVMAN 2.0 and Comprehensive Meta-analysis 2.0 [46]. The PRISMA checklist was used to check the reporting quality of the meta-analysis (Additional file 3).

## Results

### The descriptive characteristics of the studies

Eight articles met the selection criteria [29-31,47-51], which made a total of 16 independent analysis units, or groups, of which 11 were treatment groups and 5 control groups. (Additional file 2). Table 1 shows the individual characteristics of each of the integrated studies.

**Table 1:** Characteristics of included studies

**Papers (8)**	**Groups (16)**	**Study design**	**Objetive**	**Sample**	**Treatments**
Ahlqwist et al., 2008 [30]	a	Randomized controlled trial	To evaluate how 2 different treatment options affect perception of health, pain, and physical functioning over time among children and 0061dolescents with LBP	E1:23*	E1: back exercise program [(individualized physical therapy + exercise + self- training); (once a week, 12 weeks)] + back education + home exercise program (12 weeks; twice a week)
				Age: 15 (13–18)	
	b			E2:22*	E2: self-training back exercise program (1-week; 3 times a weeks; 20 mins) + back education + home exercise program (12 weeks; 3 times a week). No individualized therapy
				Age: 14 (12–17)	
Clifford, 2009 [51]	a	Prospective longitudinal cohort	To examine the clinical utility of the treatment-based classification (TBC) system by comparing treatment outcomes in a sample of children and adolescents with LBP	E1:19*	E1:Stabilization
				Age: 14.9 (12–17)	
	b			E2:11*	E2:Mobilization/Manipulation
				Age: 14.9 (12–17)	
	c			E3:4*	E3: Specific exercise
				Age: 14.9 (12–17)	
Fanucchi et al., 2009 [31]	a	Randomized controlled trial	To investigate whether exercise is effective in reducing the intensity and three-month prevalence of LBP in 12–13 year old children, compared with a control group	E:39*	E: 8-week exercise program; 8 classes, 45–60 mins each (10–15 min = educational session; 40–45 mins = exercise session) + home exercise program
				Age: 12.21 (12–13)	
	b			C:32*	C: without treatment
				Age: 12.39 (12–13)	
Fernandes et al., 2009 [50]		Case series	To evaluate the effect of a protocol of manual therapy on pain and lumbar mobility in adolescent athletes with LBP	E:18*	Protocol of therapy manual. Myofascial technique and stretching. 1 session
				Age: (15–17)	
Harringe et al., 2007 [47]	a	Clinical controlled trial	To evaluate a specific segmental muscle training program of the lumbar spine in order to prevent and reduce LBP in young teamgym gymnasts	E:15*	E: Specific muscle control exercises of the lumbar spine - the training program (8 week = week 5–12 of the study period)
				Age: 13 (11–16)	
	b			C:4*	C: without treatment
				Age: 14 (11–16)	
Jones et al., 2007 [29]	a	Randomized controlled trial	To evaluate the efficacy of a specific exercise rehabilitation program as a treatment to treat recurrent nonspecific LBP in adolescents	E:27*	E: 8-week school-based exercise programme; 16 sessions (30 mins; twice a week). Combination of strength, flexibility, and aerobic exercises + home-based exercise
				Age: 14.6 (14–15)	
	b			C:27*	C: without treatment
				Age: 14.6 (14–15)	
Perich et al., 2011 [49]	a	Clinical controlled trial	To determine whether a multi-dimensional treatment programme was effective in reducing the incidence of LBP and the associated levels of pain and disability in schoolgirl rowers	E: 33*	E: multidimensional programme [individualised exercise programme basaded on an individual musculoskeletal screening (week 1) + education session conducted by a physiotherapist (week 2) + follow up musculoskeletal screening sessions (weeks 3) + off-water-conditioning programme conducted by a Physical Education teacher (weeks 3–23)]
				Age: (14–17)	
	b			C:42*	C: without treatment
				Age: (14–17)	
Thorpe et al., 2009 [48]	a	Clinical controlled trial	To determine the efficacy of a specific physical therapy treatment administered to adolescent female rowers with the aim of decreasing the prevalence of LBP and associated levels of pain and disability	E:10*	E: education session (1 session) + physical therapy exercise treatment (3 sessions) + physical conditioning program
				Age: 13.9 (13–17)	
	b			C:8*	C: education session (1 session) + physical conditioning program
				Age: 13.8 (13–17)	

E: Experimental; C: Control; LBP: Low back pain; * Number of subjects with low back pain in the posttest.

Regarding the type of treatment, 2 treatment groups (18.18%) used exercise, 1 group (9.09%) used manual therapy, 1 group (9.09%) used the combined treatment of back education and therapeutic physical conditioning, and 7 groups (63.64%) used exercise combined with other treatments.

Regarding the quantitative treatment variables, the average number of weeks of the treatment (duration) was 12, the average number of hours per week of treatment received by each subject (intensity) was 1 hour per week, and the average total number of hours of treatment received by each subject (magnitude) was 17 hours.

As for the context variables, two qualitative variables were analyzed: the country and the place where the treatment took place. The countries most represented in this meta-analysis are Australia (25%) and Sweden (25%), followed by the United States (18.75%), South Africa (12.5%), the United Kingdom (12.5%), and Brazil (6.25%). With regard to the place where the treatment was applied, most of the studies were carried out in schools.

The subject variables analyzed were age and gender, and both were analyzed quantitatively. Specifically, the mean age was 14.1 years and the average percentage of males was 26.5%.

In regard to the quantitative methodological variables, the total sample size of subjects at the posttest was 334 subjects, specifically 221 participants in the treatment group and 113 in the control groups.

In regard to the methodological quality of the studies evaluated in 11 treatment groups, the minimum score was 2.8 and the maximum score was 6 out of a maximum of 8 points. Table 2 presents the results of the methodology evaluation. In two articles (three treatment groups) subjects were randomly assigned to groups [30,31] and in one article the groups were randomly assigned but the subjects were not [29]. Only one study [48] used an active control group, in comparison with an inactive control group. The sample size in the treatment group at the posttest was more than fifteen subjects in seven articles [29-31,47,49-51] (eight treatment groups). In three articles there was attrition in the treatment group [29,47,48] and intent-to-treat analysis was not reported, and in five articles [30,31,49-51] (eight treatment groups) all of the subjects completed the study. As to whether the assessor was masked, 2 articles [31,51] (four treatment groups) recorded this item. The evaluation of the results of all of the subjects was similar, with regards to context, time, etc. And finally, none of the studies carried out reliability analyses of the evaluation instruments or none mentioned doing so.

**Table 2:** Methodological quality of the 11 treatment groups

**Article (8)**	**TG (11)**	**1**	**2**	**3**	**4**	**5**	**6**	**7**	**8**	**Total**
Ahlqwist et al. [30]	a	1	0	1	1	1	0	1	0	5
	b	1	0	1	1	1	0	1	0	5
Clifford. [51]	a	0	0	1	1	1	1	1	0	5
	b	0	0	0.5	1	1	1	1	0	4.5
	c	0	0	0	1	1	1	1	0	4
Fanucchi et al. [31]	a	1	0	1	1	1	1	1	0	6
Fernandes et al. [50]	a	0	0	1	1	1	0	1	0	4
Harringe et al. [47]	a	0	0	1	0.833	0	0	1	0	2.833
Jones et al. [29]	a	0.5	0	1	0.871	0	0	1	0	3.371
Perich et al. [49]	a	0	0	1	1	1	0	1	0	4
Thorpe et al. [48]	a	0	1	0.5	0.588	0	0	1	0	3.088

TG: Treatment group; **1: Random assignment;** 1: the subjects were randomly assigned to the experimental conditions; 0.5: there was no random assignment but some method was applied to control confounding variables; 0: there was neither random assignment nor control of confounding variables. **2: Type of control group;** 1: active control; 0: inactive control or no control group in the design. **3: Sample size in the posttest;** 1: *N* ≥ 15 subjects; 0.5: 8 ≤ *N* < 14; 0: *N* < 8. **4: Attrition;** this is computed as 1 – attrition in the posttest. **5: Intent-to-treat analysis;** 1: present; 0: absent. **6. Evaluator blinding;** 1: present; 0: absent. **7: Homogeneous assessment;** 1: present; 0: absent. **8: Inter-rater reliability;** 1: present; 0: absent.

In terms of extrinsic characteristics, 13 of the 16 independent analysis units came from published sources. The most common profession of the first author was physiotherapist, and all of the studies were carried out between 2007 and 2011.

### Distribution of the effect sizes

Due to the large variability of the symptoms presented by children and adolescents with LBP, a meta-analysis was carried out separately for each combination of outcome measures (pain, disability, flexibility, endurance and mental health) and measurement type (self-reports and clinician assessments). Of the 11 groups treated, the type of outcome measure most commonly used was pain, in 11 groups (100%), followed by disability, in 7 groups (63.6%).

**Figure 1:**
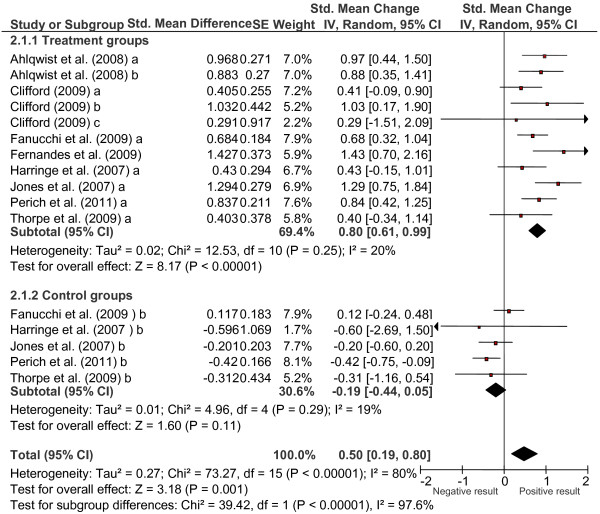
Forest plot of the standardized mean change indices calculated for the treatment and the control groups for pain outcome measures.

Table 3 presents the results of the weighted ANOVAs applied to compare the effect sizes obtained from the treated and control groups with the different outcome variables and Figure 1 shows the corresponding forest plots for pain measures. The weighted mean effect size obtained for the overall outcome measures in the 11 treated groups was *d*_+_ = 0.548 and it was statistically significant. According to Cohen’s criteria [43], we can consider this effect size to be of medium magnitude and clinically relevant. However, the 5 control groups obtained a mean effect size of *d*_+_ = −0.182, the negative sign indicating that the subjects in the control groups not only did not improve but they actually worsened slightly.

**Table 3:** Results of the weighted ANOVAs applied to compare the mean effect sizes obtained with the treatment and the control groups for the different outcome measures

			***95% C.I.***		***ANOVA***
**Outcome measure / Group**	***k***	***d***_**+**_	***d***_**l**_	***d***_**u**_	
*Pain:*					*Q*_B_(1) = 39.422, *p* < .001; *R*^2^ = .932
					*Q*_W_(14) = 17.493, *p* = .231
Treatment groups	11	.800	.611	.989	
Control groups	5	–.194	–.440	.052	
*Disability:*					*Q*_B_(1) = 6.840 *p* = .009; *R*^2^ = .561
					*Q*_W_(7) = 15.495, *p* = .030
Treatment groups	7	.661	.353	.968	
Control groups	2	–.081	–.544	.382	
*Flexibility:*					*Q*_B_(1) = 17.746, *p* < .001; *R*^2^ = 1
					*Q*_W_(5) = 4.352, *p* = .450
Treatment groups	5	.500	.306	.695	
Control groups	2	–.211	–.479	.057	
*Endurance:*					*Q*_B_(1) = 10.211, *p* = .001; *R*^2^ = 1
					*Q*_W_(2) = .256, *p* = .880
Treatment groups	3	.628	.361	.896	
Control groups	1	–.149	–.545	.245	
*Mental Health:*					*Q*_B_(1) = .254, *p* = .614; *R*^2^ = 1
					*Q*_W_(2) = 1.261, *p* = .532
Treatment groups	3	.373	.141	.605	
Control groups	1	.488	.107	.869	
*Overall-Self-reports:*					*Q*_B_(1) = 26.659, *p* < .001; *R*^2^ = .401
					*Q*_W_(14) = 15.724, *p* = .330
Treatment groups	11	.669	.498	.840	
Control groups	5	–.068	–.290	.153	
*Overall-Clinicians:*					*Q*_B_(1) = 6.960, *p* = .008; *R*^2^ = .647
					*Q*_W_(5) = 9.621, *p* = .087
Treatment groups	5	.429	.161	.698	
Control groups	2	–.212	–.606	.181	
*Overall-All combined:*					*Q*_B_(1) = 32.872, *p* < .001; *R*^2^ = 1
					*Q*_W_(14) = 11.375, *p* = .656
Treatment groups	11	.548	.394	.702	
Control groups	5	–.182	–.379	.014	

*k*: number of studies for each category. *d*_+_: mean effect size for each category. *d*_l_ and *d*_u_: lower and upper limits of the 95% confidence interval for the mean effect size in each category. *p*: probability levels for the different statistical tests. *Q*_B_: between-categories *Q* statistic. *Q*_W_: within-category *Q* statistic. *R*^2^: proportion of variance explained by the comparison between the treatment and the control groups.

The best results were obtained with the measurement of pain, with a mean effect size of *d*_+_ = 0.800. This was followed by disability reduction (*d*_+_ = 0.661), increased flexibility (*d*_+_ = 0.500), endurance (*d*_+_ = 0.628), and mental health (*d*_+_ = 0.373). All of the outcome measures of the treatments reached a statistically significant effect magnitude and a clinically relevant improvement of symptoms of the children and adolescents with LBP. By contrast, the control groups had negative mean effect sizes. The only exception was mental health measures, where both the treated and control groups showed positive mean effects, which were statistically and clinically significant.

The statistical analyses presented in Table 3 were obtained by assuming a pretest-posttest correlation coefficient of *r* = 0.5 in all of the studies. In order to check whether the value of *r* can affect the ANOVA results, these analyses were repeated twice: assuming *r* = 0.2 and *r* = 0.8. The results exhibited negligible differences in comparison to those obtained for *r* = 0.5. Another sensitivity analysis consisted in to repeat the ANOVAs using the REML estimator of the residual heterogeneity variance in place of that based on the method of moments. This change did not affect the meta-analytic results. The ANOVA results obtained for *r* = 0.2 and 0.8 as well as those for the REML variance estimator are not presented in this paper, but they can be obtained from the corresponding author upon request.

As the within-group effect sizes are prone to offer biased estimates of the treatment effect, with the five studies that included control groups we calculated between-groups effect sizes. Table 4 presents the mean between-groups effect sizes for the different outcome measures, as well as the difference between the treatment and control within-group means (last column) reported in Table 3. As Table 4 shows, the mean between-groups and within-group effect sizes were very similar for measures of pain (1.026 and 0.994, respectively), flexibility (0.688 and 0.711), endurance (0.870 and 0.777), mental health (−0.243 and −0.115), self-report measurements (0.628 and 0.737), clinicians assessments (0.552 and 0.641), and all measures combined (0.622 and 0.730). The main discrepancy was found in disability outcomes, where the mean between-groups effect size was clearly lower (0.359) than the mean within-group one (0.742).

**Table 4:** Results of the meta-analyses taking the between-group standardized mean differences as the effect size

**Outcome measure**	***k***	***d***_**+**_	**95% C. I.**	***d***_**T**_**- *d***_**C**_
			***d***_**l**_***d***_**u**_	
Pain	5	1.019	0.478 1.561	0.994
Disability	2	0.366	−0.345 1.077	0.742
Flexibility	2	0.703	−0.024 1.430	0.711
Endurance	1	0.870	−0.212 1.952	0.777
Mental health	1	−0.243	−1.235 0.749	−0.115
Self-reports	5	0.656	0.127 1.184	0.737
Clinicians	2	0.548	−0.176 1.273	0.641
Combined	5	0.664	0.139 1.189	0.730

*k*: number of studies for each outcome measure. *d*_+_: mean effect size for each outcome. *d*_l_ and *d*_u_: lower and upper limits of the 95% confidence interval for the mean effect size in each outcome. *d*_T_ – *d*_C_: difference between the treatment and control within-group means (calculated from Table 3). All of the heterogeneity *Q* statistics were not statistically significant (*p* > .05) and all *I*^2^ indices were equal to 0%.

### Analysis of publication bias

Because 18.75% of the independent analysis units were unpublished, we checked whether publication bias could be a threat against our meta-analytic results. To do this, we calculated the Egger test [44].

The tests were carried out for just the two dependent variables with a reasonable number of data: pain (*k* = 11 treated groups) and disability (*k* = 7). In both cases the Egger test did not reach a statistically significant result (*p* = .837 and *p* = .390, respectively). Therefore, we can discard publication bias as a threat to our meta-analytic results.

### A predictive model

In order to estimate the differential effectiveness of the treatments, and because the studies combined different treatment techniques, we applied a multiple meta-regression model in which we proposed a predictive model that allowed us to determine the relative effectiveness of the treatments. To this end, we defined by means of dummy coding (0, absent; 1, present) four dichotomous predictors which represented the four types of treatment used most frequently in the studies: exercise, back education, therapeutic physical conditioning and manual therapy. The results are presented in Table 5. As shown, the model was statistically significant (*p* = .012) and had a percentage of explained variance of 57.3%. However, none of the four types of treatment was statistically significant once the influence of the other treatments was partialized out. The predictive equation was: d’ = 0.0107 + 0.283*Exercise + 0.031*Back education + 0.536* Therapeutic physical conditioning + 0.508*Manual therapy. This predictive equation enabled us to estimate the effects of different types of treatment, alone or in combination. Thus, for example, the estimated effects when using each type of treatment in isolation are 0.294 for exercise, 0.042 for back education, 0.547 for therapeutic physical conditioning and 0.519 for manual therapy. The most effective combination was that of therapeutic physical conditioning and manual therapy, with an estimated effect of 1.055.

**Table 5:** **Results of the mixed-effects multiple meta-regression to examine the differential effectiveness of the treatments (*k*** **= 16)**

**Predictor**	***b***_***j***_	***SE***	***Z***	***p***
Exercise	.283	.267	1.059	.289
Back education	.031	.329	.093	.926
Therapeutic physical conditioning	.536	.326	1.643	.100
Manual therapy	.508	.286	1.777	.075
Full model: *Q*_R_ (4) = 12.842, *p* = .012; *R*^2^ = .573				
*Q*_E_ (11) = 27.578, *p* = .004				
Predictive equation: *d’* = 0.0107 + 0.283*Exercise + 0.031*Back education + 0.536* Therapeutic physical conditioning + 0.508*Manual therapy.				

*k*: number of studies. *b*_*j*_*:* regression coefficient. *SE:* standard error of the regression coefficient*. Z*: Z test for examining the significance of each treatment*. p:* probability level associated to the corresponding statistical test. *Q*_R_: statistic to examine the significance of the full model. *Q*_E_: statistic to assess the misspecification of the full model. *d*^’^: predicted effect size.

## Discussion

In this paper we have presented the results of a meta-analytic study on the effectiveness of physical therapy treatments for LBP in children and adolescents. With this objective, eight articles met the selection criteria, which allowed us to define 11 treatment groups and 5 control groups. For each group of participants, the effect size index was defined as the standardized mean change between the pretest and posttest. With the five studies that included a control group, between-groups effect sizes were also calculated. In order to obtain all of the possible changes due to the treatments, we calculated an effect estimate for each of the outcome measures (pain, disability, flexibility, endurance and mental health) and the measurement type (self-reports and clinician assessments).

According to Cohen [43], the mean effect size obtained with the treatment groups for the different outcomes measures achieved effect magnitudes ranging from low-medium to high, all being statistically significant.

The control groups, however, obtained negative mean effects that were not statistically significant. In addition, the comparison between the mean effects of the treated and control groups was statistically significant in favour of the treated groups. The only exception to this pattern of results occurred with mental health measures, where the mean effects of both the treated and the control groups were positive, statistically significant and of medium-low magnitude. With the purpose of checking whether the within-group effect sizes were overestimating the treatment effects, for each outcome measure the mean between-groups effect size was also calculated, finding very similar results, in general, to those obtained with the within-study effect sizes. Therefore, we can conclude that the within-group effect sizes are not overestimating the true effect in the population.

Although our intention was to analyze the treatment outcomes in the posttest and follow-up, this was not possible because only one article included follow-up data [31].

To test whether different types of treatment offer differential benefits, we applied a multiple meta-regression model, whose results enabled us to confirm the initial hypothesis that there are differences in effectiveness between the treatments based on exercise, education, therapeutic physical conditioning and manual therapy, the combination of the latter two being the most promising treatment.

Our study has, however, some limitations that reduce the generalizability of our results. Firstly, the base of meta-analyzed studies is very small, with only 16 units of analysis collected from eight articles. Under these conditions, the use of inferential techniques such as ANOVA or meta-regression to find moderating variables that influence the effectiveness of treatments severely limits the scope of our results. Therefore, the results should be interpreted cautiously pending further studies of similar treatments. The small number of meta-analyzed studies led us to dramatically reduce the analysis of moderating variables that we had coded, restricting our moderator analysis exclusively to treatment types. Secondly, the lack of control groups forced us to change the unit of analysis and, consequently, the effect size index. Instead of comparing the means of the treated and control groups using the standardized mean difference, we had to define the group as our unit of analysis and use as the effect size the standardized mean change index, which is methodologically weaker than the former effect size. It is hoped that future studies incorporate control groups in order to obtain more valid estimates of treatment effects.

### Implications for clinical practice

Our results show that physical therapy treatments seem to be effective for LBP in children and adolescents, with the combination of physical therapy conditioning and manual therapy showing the best results. There is no evidence regarding the duration of the beneficial effects of these treatments, because the studies do not provide follow-up information.

### Considerations for future research

The results of our meta-analysis enable us to propose some recommendations for future research in this field. It would be advisable for future studies to specify in the greatest possible detail the aspects of the treatments applied, such as intensity, duration and magnitude. If this is not done, it is not possible to assess the benefits that each type of treatment is able to give to children and adolescents. Another aspect that should be improved in the design of these studies is the inclusion of follow-up data for the treatment and control groups. It should also be noted that the results of research on the effectiveness of physical therapy in children and adolescents with LBP have serious methodological flaws in their design, implementation and data analysis. Assignment of subjects to groups should be random, masked assessors should be used and intent-to-treat analyses should be carried out. All of these measures will enable us to have greater control of potential biases in the treatment effect estimates.

## Conclusion

In conclusion, of all the physical therapy treatments for LBP in children and adolescents, the combination of therapeutic physical conditioning and manual therapy appears to be the most effective. However, the low number of studies and control groups, and the methodological limitations in this meta-analysis prevent us from drawing definitive conclusions in relation to the efficacy of physical therapy treatments in LBP.

## Competing interests

The authors declare that they have no competing interests.

## Authors’ contributions

All authors contributed to conception and design, acquisition, analysis and interpretation of data and drafting of the manuscript. AGC and JSM participated in the critical revision of the manuscript for important intellectual content. ICM and JSM performed statistical analyses. All authors read and approved the final manuscript.

## Pre-publication history

The pre-publication history for this paper can be accessed here:

http://www.biomedcentral.com/1471-2474/14/55/prepub

## Supplementary Material

Additional file 1 Search strategy for Medline. Click here for file

Additional file 2 Flow diagram of the identification and selection of studies for inclusion. Click here for file

Additional file 3 PRISMA Checklist. Click here for file
