# Supplementary material for: Physical therapy treatments for low back pain in children and adolescents: a meta-analysis
Source: BMC Musculoskelet Disord. 2013 Feb 2;14:55. doi: 10.1186/1471-2474-14-55 (PMC3568715; doi:10.1186/1471-2474-14-55)
Supplement: Additional file 1 — Search strategy for Medline. [file 1471-2474-14-55-S1.doc]

|  | | | |
| --- | --- | --- | --- |
| **Additional file 1**  Search strategy for Medline | | | |
| **Key words** |  | |  |
| 1. Adolescent* 2. Child* 3. Youn* 4. School* 5. 1 or 2 or 3 or 4 6. Back pain 7. Low back pain 8. Back complaint* 9. Back care 10. 6 or 7 or 8 or 9 11. 5 and 10 12. Treatment 13. Intervention 14. Education 15. Postural hygiene | 1. Posture education 2. Back function 3. Physiotherapy 4. Ergonomics 5. Physical therapy 6. Exercise 7. Exercise therapy 8. Management 9. Chiropractic 10. Physical fitness 11. Movement techniques 12. Acupuncture 13. Tens 14. Massage 15. Spinal manipulation | 1. Rehabilitation 2. Back school 3. Conservative 4. Manual therapy 5. Recuperation 6. 12 or 13 or 14 or 15 or 16 or 17 or 18 or 19 or 20 or 21 or 22 or 23 or 24 or 25 or 26 or 27 or 28 or 29 or 30 or 31 or 32 or 33 or 34 or 35 7. 11 and 36 |  |
| **Options:** all years, all languages, all publication types, all citation subsets, all child (0-18 years),  species (humans), all genders, all databanks, all statues. | | | |
| **Field Tags:**TS=Topic* |  |  |  |
|  | **Results: 525** |  |  |
