# Supplementary material for: Physical therapy treatments for low back pain in children and adolescents: a meta-analysis
Source: BMC Musculoskelet Disord. 2013 Feb 2;14:55. doi: 10.1186/1471-2474-14-55 (PMC3568715; doi:10.1186/1471-2474-14-55)
Supplement: Additional file 2 — Flow diagram of the identification and selection of studies for inclusion. [file 1471-2474-14-55-S2.doc]

| **Additional file 2** |
| --- |
| Flow diagram of the identification and selection of studies for inclusion |

Records identified by searching databases Cochrane Library (255), ISI Web of Knowledge (520), Medline (525), PEDro (29), LILACS (8)

(n = 1337)

Additional records identified via other sources

(n = 35)

Records screened

(n = 1372)

Records after removal of duplicates

(n = 1372)

Full-text articles

assessed for eligibility

(n=44)

Full-text articles excluded

(n=36)

> 18 years (n=19)

Preventive treatments (n=3)

Absence statistical data (n=4)

Other pain (n=7)

Spinal deformities (n=3)

Articles included in the quantitative

synthesis (meta-analysis)

(n=8):

5 articles (1 experimental group and 1 control group)

1 article (2 experimental groups)

1 article (3 experimental groups)

1 article (1 experimental group)

Studies included in the quantitative synthesis (meta-analysis)

(n=16)

Identification

Records excluded

(n = 1328)

Screening

Eligibility

Included
